# Morphology and physiology of olfactory neurons in the lateral protocerebrum of the silkmoth *Bombyx mori*

**DOI:** 10.1038/s41598-019-53318-8

**Published:** 2019-11-12

**Authors:** Shigehiro Namiki, Ryohei Kanzaki

**Affiliations:** Research Center for Advanced Science and Technology, The University of Tokyo, 4-6-1 Komaba, Meguro, 153-8904 Tokyo Japan

**Keywords:** Sensory processing, Animal physiology

## Abstract

Insect olfaction is a suitable model to investigate sensory processing in the brain. Olfactory information is first processed in the antennal lobe and is then conveyed to two second-order centres—the mushroom body calyx and the lateral protocerebrum. Projection neurons processing sex pheromones and plant odours supply the delta area of the inferior lateral protocerebrum (∆ILPC) and lateral horn (LH), respectively. Here, we investigated the neurons arising from these regions in the brain of the silkmoth, *Bombyx mori*, using mass staining and intracellular recording with a sharp glass microelectrode. The output neurons from the ∆ILPC projected to the superior medial protocerebrum, whereas those from the LH projected to the superior lateral protocerebrum. The dendritic innervations of output neurons from the ∆ILPC formed a subdivision in the ∆ILPC. We discuss pathways for odour processing in higher order centres.

## Introduction

Owing to its relative simplicity compared with vertebrates, insect olfaction is a suitable model to investigate sensory processing in the nervous system. The organization of olfactory processing is less complex in insects. Two-layered networks process odorants detected by the antennae: initial processing occurs at the antennal lobe (AL), and the information is conveyed further on to higher brain centres, including the calyx of the mushroom body (MB) and the lateral protocerebrum (LPC), for second-order processing. These circuits are connected by second-order neurons called the projection neurons (PNs), which run through multiple pathways^1–3^. Each pathway exhibits unique features, such as the travelling path, targeting area, commissural innervation, and mode of odour processing^1,4–9^. The MB is considered a centre for learning and memory, and downstream neurons of the MB have been well characterized^10,11^. Because the spatial map in the LPC is innately programmed, the LPC is thought to dictate experience-independent olfactory behaviour^12,13^. However, the olfactory pathway from the LPC and the property of the output neurons from the LPC have yet to be revealed.

In the present study, we conducted population labelling of LPC output neurons (third-order olfactory neurons) and intracellular recording with a sharp glass microelectrode to identify individual neurons that receive the AL output in the brain of a silkmoth, *Bombyx mori*. The LPC is further divided into the delta area in the inferior lateral protocerebrum (∆ILPC) and the lateral horn (LH). In an earlier study using *B. mori*, we reported that PNs processing major pheromone components send their axons to the ∆ILPC^2,14^, which is in turn connected with a higher order centre, the superior medial protocerebrum (SMP)^15^. In addition, we reported that a major portion of other PNs that process plant odour information send their axon to the LH^2,14^. Here, we examined population-level anatomy using confocal microscopy and characterized the LPC output neurons and their projection in more detail by intracellular recording and immunostaining and found that ∆ILPC and LH supply different areas in the protocerebrum.

## Materials and Methods

### Experimental animals

Male silkworm moths, *B. mori* L. (Lepidoptera: *Bombycidae*), were reared in the laboratory. The larvae of moths were reared from eggs in the laboratory on an artificial diet (Silk Mate 2S; Nosan Bio Department, Yokohama, Japan) at 26 °C and 60% relative humidity under a 16 h light/8 h dark photoperiod. Moths were used within 2–7 days after eclosion.

### Intracellular recording and staining

Moths were anaesthetized by cooling. The abdomen, legs, wings, and dorsal side of the thorax were removed. Each moth was fixed in a chamber, and its head was immobilized using a notched plastic yoke slipped between the head and thorax. The brain was exposed by opening the head capsule and removing the large tracheae. The intracranial muscles were removed to eliminate brain movement. The dorsal AL was surgically desheathed to facilitate the insertion of a glass microelectrode (TW100F-3; World Precision Instruments, FL) by using a commercial fibre puller (P2000; Sutter Instruments, CA). Electrodes were filled with 5% Lucifer Yellow CH (LY) fluorescent dye solution (Sigma, St Louis, MO) in 0.1 M LiCl or distilled water. The resistance of the electrodes was approximately 150 MΩ. A silver ground electrode was placed on the head cuticle, and the brain was superfused with a saline solution comprising 140 mM NaCl, 5 mM KCl, 7 mM CaCl_2_, 1 mM MgCl_2_, 4 mM NaHCO_3_, 5 mM trehalose, 5 mM N-tris (hydroxymethyl) methyl-2-aminoethanesulfonic acid, and 100 mM sucrose (pH 7.3). The electrodes were inserted using a micromanipulator (Leica Microsystems, Wetzlar, Germany). The signals were amplified (MEZ-7200; Nihon Kohden, Tokyo, Japan), monitored with an oscilloscope (VC-9; Nihon Kohden, Tokyo, Japan), and recorded on digital audiotapes (RD-125T; TEAC, Tokyo, Japan) at 24 kHz. The acquired signals were converted using an analogue-to-digital converter and stored on a computer (Quick Vu 2; TEAC, Tokyo, Japan). We stained each neuron using an iontophoretic injection of LY and a constant hyperpolarizing current (from approximately −1 to −5 nA) for 1–3 min. The brain was fixed for 4–10 h at room temperature in 4% paraformaldehyde in 0.1 M phosphate buffer solution (pH 7.3) containing 10% sucrose, dehydrated in ethanol, and cleared in methyl salicylate.

### Population staining

For mass staining, the brain was dissected from the head capsule and transferred to a small chamber containing a pool created using paraffin wax (Soft plate wax; GC, Tokyo, Japan) on a cover glass (24 × 60 mm; C024601, Matsunami, Japan). The pool was filled with saline solution as described above. The chamber was placed under a fixed-stage upright microscope (BX51WI; Olympus, Tokyo, Japan) equipped with differential interference contrast optics and long-working-distance objectives (×40 LUMPlanFL/IR water immersion; Olympus, Tokyo, Japan). The image was monitored using a charge-coupled device camera (C2741-79; Hamamatsu Photonics, Shizuoka, Japan). To investigate the olfactory pathway in the brain, we conducted mass staining of the neuronal population with dye injection into the ∆ILPC and LH and characterized the brain regions connected. A glass microelectrode (20–50 MΩ) filled with 4% LY solution or 10% tetramethylrhodamine dextran (TMR) (D3308; Molecular Probes, Eugene, OR) was placed dorsally to the AL in the brain. The dye was injected iontophoretically at a pulse rate of 5 Hz for 40–100 ms and a 100–700-nA hyperpolarizing pulse current for LY and a depolarizing pulse for TMR using an electric stimulator (SEN-7203), isolator (SS-202J), and amplifier (MEZ-8300; Nihon Kohden, Tokyo, Japan). The total injection time was 10–30 min. The preparations were stored at 4 °C for 4 h to allow the dye to diffuse, and the brains were dissected from the heads. After washing with 0.1 M phosphate buffer solution, the brains were fixed for 1–24 h with 4% paraformaldehyde, dehydrated with ethanol, and cleared with methyl salicylate.

### Olfactory stimulation

Two synthetic principal pheromone components of *B. mori*, namely, (E,Z)-10,12-hexadecadien-1-ol (bombykol) and (E,Z)-10,12-hexadecadien-1-al (bombykal), with >99% purity (confirmed by gas chromatography), were dissolved in HPLC-grade n-hexane (Butenandt *et al*. 1959; Kaissling *et al*. 1978). We applied 10 ng of bombykol to the filter paper. We used 8.48 µg of the leaf alcohol *cis*-3-hexen-1-ol (purity >97%, #H0124; Tokyo Chemical Industries, Tokyo, Japan) as the principal host plant odour. In addition, we used 8.46 µg of trans-2-hexenal (purity >98%, 13265–9; Sigma, St Louis, MO) and a racemic mixture of 8.62 µg of linalool (purity >98%, #L0048; Tokyo Chemical Industries, Tokyo, Japan) and 8.88 µg of citral (purity >93%, D0762; Tokyo Chemical Industries, Tokyo, Japan). Plant odorants were dissolved in mineral oil (76235; Fluka, Buchs, Switzerland). Furthermore, 5 µL of the odorant solution was applied to a piece of filter paper (1 × 2 cm) and inserted into a glass stimulant cartridge (5.5 mm tip diameter). The distance between the filter paper and the exit of the cartridge was approximately 7 cm. Odour concentration was expressed as the amount of stimulant applied to the filter paper. Further, an odour stimulus was applied to the antennae ipsilateral to the hemisphere impaled by the microelectrode, and the exit of the cartridge was positioned 1.5 cm from the antennae. Compressed, clean air was passed through a charcoal filter into the stimulant cartridge, and each stimulus was applied at a velocity of 500 mL/min (approximately 35 cm/s). The stimulus duration was usually 500 ms, and the interval between puffs was at least 10 s. An exhaust tube was placed on the opposite side of the stimulant cartridge to remove odours (inner diameter, 4.5 cm; 15 cm from the antennae; approximately 55 cm/s). All stimulant cartridges were sealed with a Teflon sheet and stored at −20 °C before use and brought to room temperature prior to a recording session. We used solenoid valves (MTV-31-M6; Takasago Electric, Nagoya, Japan) and an interface board (Digidata 1200; Axon Instruments, Foster City, CA) to control stimulus cartridge selection, stimulus duration, and intervals with a customized programme written in BASIC.

### Confocal microscopy

Each stained neuron was imaged frontally using a confocal imaging system (LSM510; Carl Zeiss, Jena, Germany) with a plan apochromat × 40 (numerical aperture = 1.0) objective. Whole mounts of the LY-stained neurons were examined at 458-nm excitation wavelength with a long-pass emission filter (>475 nm). Serial optical sections were acquired at 0.7- or 1.4-μm intervals through the entire depth of the neuron. Furthermore, three-dimensional reconstructions of the labelled neurons were created from these sections. We identified ∆ILPC by its relatively higher autofluorescence than other regions in the protocerebrum and its characteristic pyramidal shape (Seki *et al*., 2005). In some cases, autofluorescence signals of the tissue were examined at an excitation wavelength of 543 nm (He–Ne laser) with a long-pass emission filter (>560 nm) in whole mounts.

Image contrast and brightness were modified using ImageJ (National Institutes of Health, Bethesda, MD). The figures were prepared in Adobe Illustrator CS (Adobe Systems, San Jose, CA). Colour images were used for the neuron data obtained with an autofluorescence signal.

## Results

We examined mass staining by injecting dye into the LPC and identified candidate downstream areas. We then obtained intracellular recordings and identified the morphology of seven neurons whose innervations were matched to the pattern obtained by mass staining.

### Areas connected with the ∆ILPC identified by mass staining

**Figure 1:**
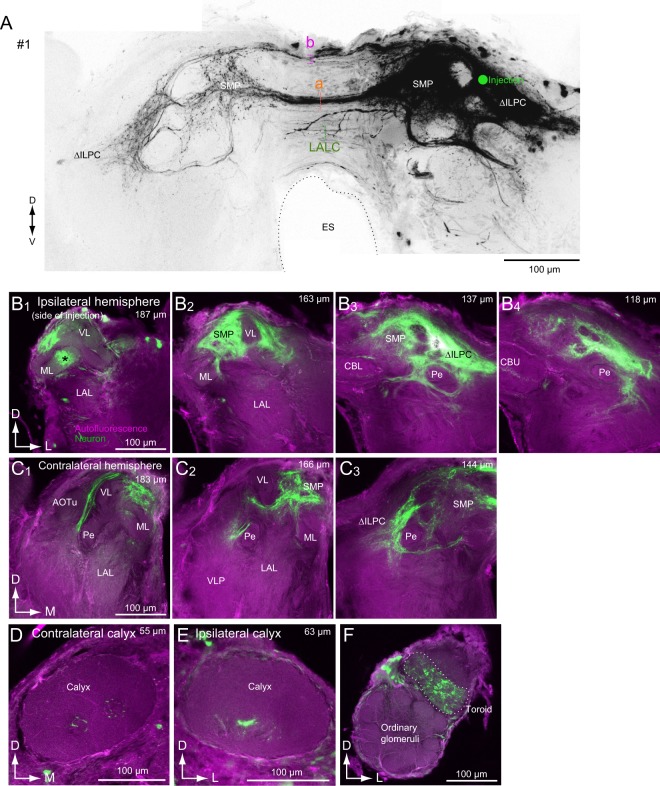
Mass staining into the delta area of the inferior lateral protocerebrum (ΔILPC). (**A**) Maximum-intensity projection of the mass staining results. The green circle represents the dye-injection site in the ΔILPC. The original data are taken from^15^. (**B**) Confocal stacks of the right hemisphere in the mass staining sample shown in (**A**). Green shows Lucifer yellow signals in stained neurons, and magenta shows the autofluorescence of the tissue. Depth from the posterior surface is shown in the top-right panel. The superior medial protocerebrum (SMP) was densely labelled. (**C**) Confocal stacks of the left hemisphere in the mass staining sample shown in (**A**). The SMP and ΔILPC were labelled. (**D,E**) Calices in contralateral (**D**) and ipsilateral hemispheres are shown (**E**). The ventral part is labelled. (**F**) The antennal lobe in the ipsilateral hemisphere. The shape of the toroid glomerulus is shown with a broken line. The innervation was confined within the toroid. AOTu, anterior optic tubercle; CBL, central body lower division; CBU, central body upper division; ES, oesophagus; ML, medial lobe of the mushroom body; LAL, lateral accessory lobe; Pe, pedunculus of the mushroom body; VL, vertical lobe of the mushroom body; VLP, ventral lateral protocerebrum.

**Figure 2:**
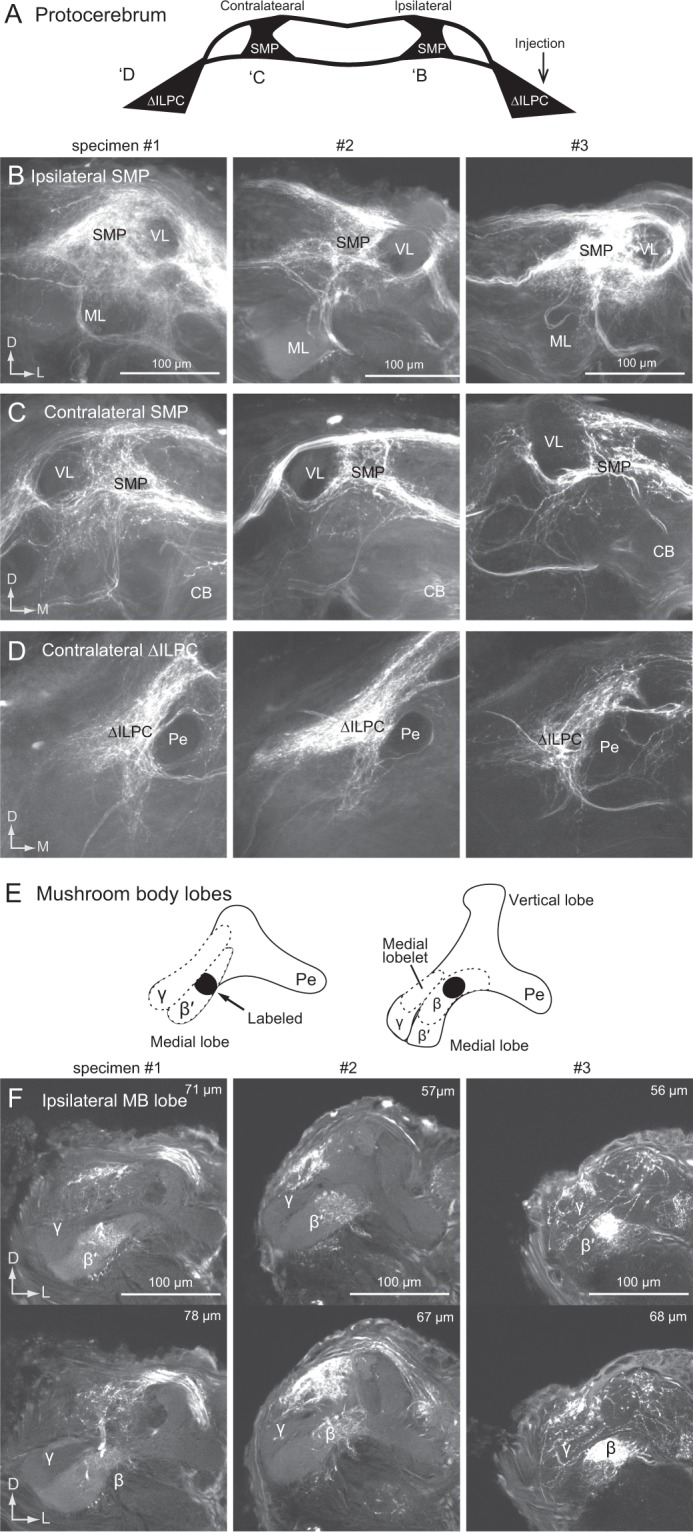
Consistency of mass staining results following dye injection into the delta area of the inferior lateral protocerebrum (ΔILPC). (**A**) Schematics of the dye injection results in the protocerebrum. Regions densely connected to the ΔILPC are shown. The number corresponds to panels in B-D. (**B–D**) Maximum-intensity projection images for the area of the superior medial protocerebrum (SMP) in the ipsilateral hemisphere and the SMP (**C**) and ΔILPC (**D**) in the contralateral hemisphere. Staining samples from three specimens are shown. (**E**) Summary of the staining result of mushroom body lobes. The shallow (left) and deep areas of the mushroom body lobes are shown (right). The staining was observed in the β and β′ lobes (black). (**F**) Confocal stacks of the anterior medial part of the protocerebrum in the hemisphere ipsilateral to the injection site. Three different specimens are shown. The depth from the anterior surface of the protocerebrum is shown in the top-right panel. The innervation was confined within the β′ and β lobes of the mushroom body. The areas of innervation were similar among specimens. No innervation was observed in the γ and vertical lobes. CB, central body; MB, mushroom body; ML, medial lobe of the mushroom body; Pe, pedunculus of the mushroom body; VL, vertical lobe of the mushroom body.

We have reported that the dye injection in the medial portion of the ∆ILPC labels the SMP on both sides and the ∆ILPC on the contralateral side^15^ (Fig. 1). In the present study, we reinvestigated the anatomical result (Fig. 1B–F) and performed additional mass staining in the same condition as the previous study (Fig. 2). The pattern of innervation was conserved across three specimens examined (Fig. 2). Most of the SMP on the same side of the dye injection was densely labelled (Figs 1B and 2B). The innervation was biased more towards the anterior side than the posterior side (Fig. 1B). Labelling was also observed in the SMP and ∆ILPC hemisphere contralateral to the injection site (Figs 1A and 2C,D). Labelling in the SMP on the contralateral side was also biased towards the anterior side (Fig. 1C). No labelling was observed in the MB lobes in the contralateral hemisphere (Fig. 1C), and the entire field in the ∆ILPC was labelled (Fig. 1C3). The central zone in the MB calyx was labelled on both sides (Fig. 1D,E). The toroid glomerulus was labelled in the ipsilateral hemisphere (Fig. 1F), which was consistent with the anatomical profile of PNs^2^.

Two major commissural pathways running through the anterior brain towards the contralateral hemisphere were observed (Fig. 1A). One pathway ran dorsal to the central body and innervated the bilateral SMP (Fig. 1A, “a”), whereas the other pathway ran along the dorsal edge of the brain and innervated the bilateral ∆ILPC (Fig. 1A, “b”). In addition, few neurons ran through the lateral accessory lobe commissure (LALC) (Fig. 1A), from which some additional branches invaded the SMP.

We analysed the innervation of the medial lobe of the MB in more detail (Fig. 2E,F). Dye injection into the ∆ILPC labelled a portion of the medial lobe, whereas innervation was not observed in the vertical lobe, Y-lobe, and pedunculus. MB lobes are further divided into several lobelets. Among these, the β and β′ lobelets were partially labelled, whereas the γ and medial lobelets were not labelled. This staining pattern was conserved across three specimens, indicating a connection between the ∆ILPC and MB lobes.

### ∆ILPC neurons

We obtained a few stained neurons that innervated the SMP and ∆ILPC. To characterize the individual neuron morphology that constitutes the neuronal pathway identified by mass staining, we performed the intracellular recording and staining of neurons innervating the ∆ILPC. However, the ∆ILPC is located deep in the brain, which makes recording difficult. Mass staining revealed that the anterior SMP was well connected to the ∆ILPC. We therefore inserted electrodes into the SMP to record neurons connecting the ∆ILPC and SMP. This approach efficiently recorded neurons innervating the ∆ILPC. The basic morphological features of five neurons are summarized in Table 1.

**Table 1:** Characteristics of intracellular staining.

Cell type	Figure	Innervation side	Smooth process*	Blebby process*
∆ILPC–SMP BN	Fig. 3	bilateral	∆ILPC (whole), type-I	SMP, ∆ILPC
∆ILPC–SMP UN	Fig. 4	unilateral	∆ILPC (whole), type-I	SMP
∆ILPC–MB UN	Fig. 5	unilateral	∆ILPC (lateral), type-II	Calyx
∆ILPC–MB BN	Fig. 6	bilateral	∆ILPC (lateral), type-II	Calyx
MB–SMP–∆ILPC UN	Sfig. 1	unilateral	MB lobelets	SMP, ∆ILPC
LH–SLP BN #1	Fig. 8	bilateral	LH (whole)	SLP
LH–SLP BN #2	Fig. 8	bilateral	LH (anterior)	SLP
LH–MB UN	Fig. 8	unilateral	LH (posterior)	Calyx

*Major innervation sites are shown.

#### ∆ILPC to SMP

**Figure 3:**
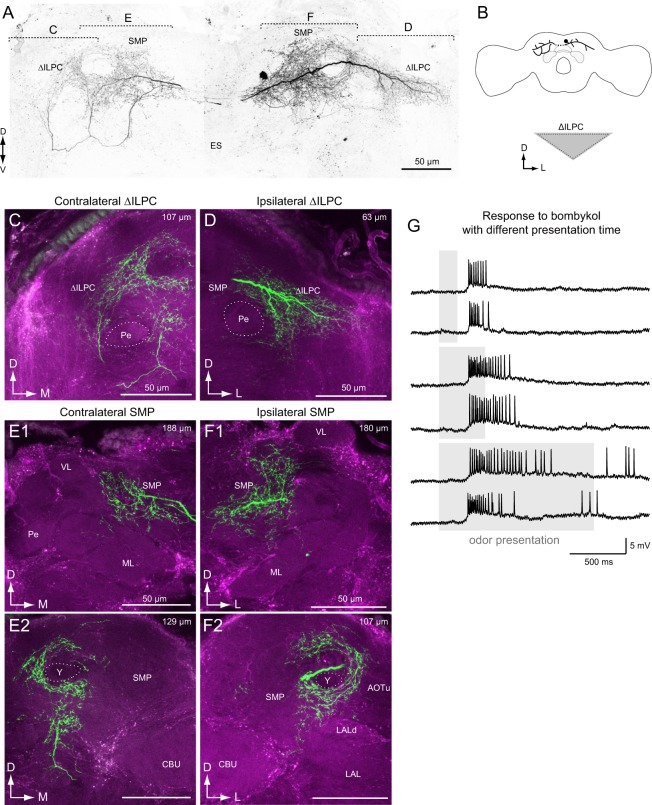
A projection neuron from the delta area of the inferior lateral protocerebrum (ΔILPC–SMP bilateral neuron). (**A**) Frontal view of the neuronal morphology. The neuron innervates the ΔILPC and superior medial protocerebrum (SMP) in both hemispheres. Green shows Lucifer yellow signals in stained neurons, and magenta shows the autofluorescence of the tissue. (**B**) Schematics of neuronal innervation. (**C**) Confocal stack of the ΔILPC in the contralateral hemisphere. (**D**) Confocal stack of the ΔILPC in the ipsilateral hemisphere. (**E**) Confocal stack of the SMP in the contralateral hemisphere. The neuron innervates the anterior SMP. (**F**) Confocal stack of the SMP in the ipsilateral hemisphere. (**G**) Response to bombykol of different time durations. The grey box represents the time period of odour presentation. AOTu, anterior optic tubercle; CBU, central body upper division; ES, oesophagus; ML, medial lobe of the mushroom body; LAL, lateral accessory lobe; Pe, pedunculus of the mushroom body; VL, vertical lobe of the mushroom body; Y, Y-lobe of the mushroom body.

A few neurons innervated the SMP. In both cases, the dendritic field covered the entire ∆ILPC, and an excitatory response to bombykol, a sex pheromone of the silkmoth, was observed. Figure 3 shows the morphology and physiology of a bilateral interneuron innervating the ∆ILPC, SMP, and inferior clamp bilaterally (∆ILPC–SMP bilateral neuron). The cell body was located on the posterior surface near the midline, and the axon ran through a commissural pathway (Fig. 1A, “a”). The innervation in the ∆ILPC in the contralateral hemisphere had a blebby appearance (Fig. 3C). Moreover, innervation with a blebby appearance was predominant in the bilateral SMP (Fig. 3D,E), suggesting information flow from the ∆ILPC to the SMP. The innervation in the ∆ILPC in the ipsilateral hemisphere had a smooth appearance (Fig. 3F). It exhibited excitation in response to bombykol (Fig. 3G). The excitatory response was transient even when applied for a long period of time (Fig. 3G, bottom). There is a possibility that this neuron does not report the duration but the onset of pheromone input, though more sampling is required for confirmation.

**Figure 4:**
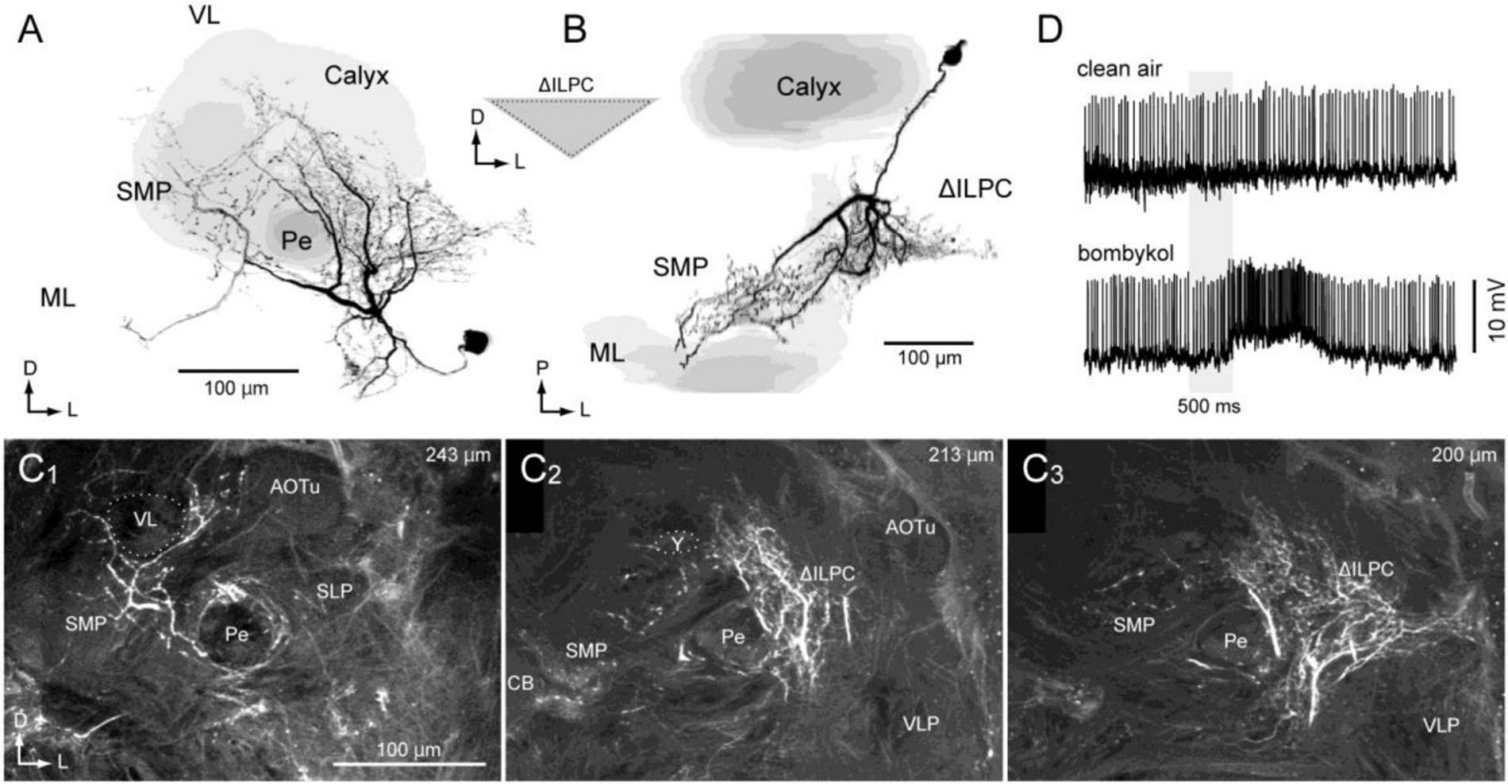
A projection neuron innervating the delta area of the inferior lateral protocerebrum and superior medial protocerebrum (ΔILPC–SMP unilateral neuron). (**A,B**) Reconstruction of the neuron (black) and mushroom body (grey). Frontal (**A**) and dorsal views are shown (**B**). The innervation in the ΔILPC and SMP with smooth and blebby appearance. (**C**) Confocal stacks of the protocerebrum. Images at three different depths are shown. (**D**) Response to clean air and bombykol. AOTu, anterior optic tubercle; ML, medial lobe of the mushroom body; LAL, lateral accessory lobe; Pe, pedunculus of the mushroom body; SLP, superior lateral protocerebrum; VL, vertical lobe of the mushroom body; VLP, ventral lateral protocerebrum.

A unilateral neuron connecting the ∆ILPC to the SMP was also identified (Fig. 4, ∆ILPC–SMP unilateral neuron). The cell body was located beside the MB calyx. The interneuron innervated the entire field in the ∆ILPC, which had a smooth appearance, whereas the SMP had a blebby appearance (Fig. 4C). The innervation in the SMP was biased towards the anterior-lateral side. The interneuron exhibited excitation in response to bombykol (Fig. 4D).

#### ∆ILPC to MB

**Figure 5:**
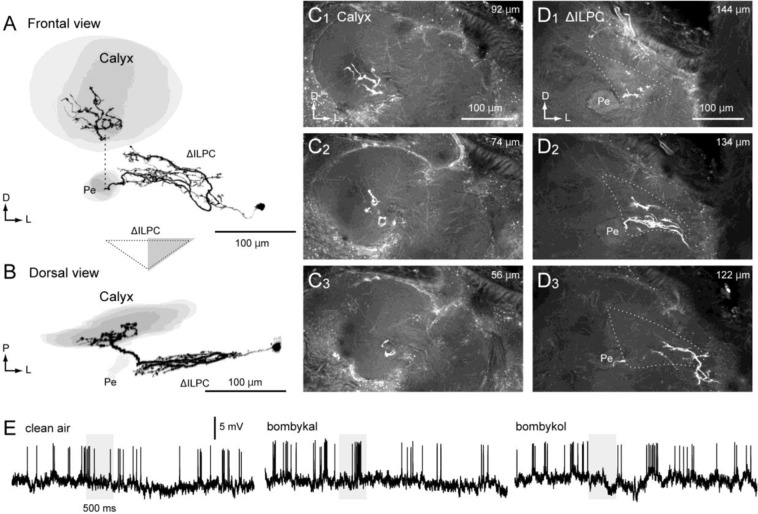
A projection neuron connecting the delta area of the inferior lateral protocerebrum and the mushroom body calyx (ΔILPC–MB unilateral neuron). (**A,B**) Frontal views of reconstruction of the neuron (black) and mushroom body (grey). Frontal (**A**) and dorsal views are shown (**B**). The innervation in the ΔILPC and SMP with smooth and blebby appearance. (**C**) Neuronal innervation in the mushroom body calyx. The innervation is biased towards the central zone of the calyx. (**D**) Neuronal innervation in the ΔILPC. Confocal stacks at three different depths are shown. The depth from the posterior brain surface is shown in the top-right panel. The innervation is biased towards the lateral ΔILPC. (**E**) Response profile of the neuron. The neuron exhibits a brief excitatory response to bombykal. Pe, pedunculus of the mushroom body.

A few neurons innervated the MB calyx. In both cases, the dendritic field was biased towards the lateral side within the ∆ILPC, showing an excitatory response to bombykal. Figure 5 shows the morphology and physiology of a unilateral interneuron (∆ILPC–MB unilateral neuron). The cell body was located in the cell cluster between the midbrain and the optic lobe. The innervation with a smooth appearance was predominant in the ∆ILPC (Fig. 5C). Notably, the innervation was biased towards the lateral side (Fig. 5D), which receives axonal projection from the cumulus glomerulus^14^. The axon ran along the peduncle and projected to the MB calyx. The innervation in the calyx had a blebby appearance and was concentrated in the central zone. The interneuron exhibited brief excitation in response to the secondary pheromone component bombykal^16^ but not to bombykol (Fig. 5E).

**Figure 6:**
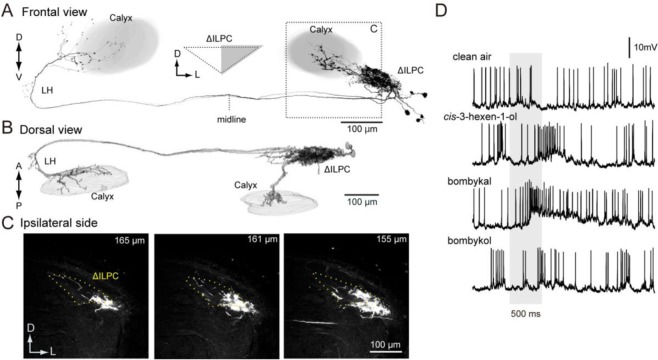
Neurons innervating the delta area of the inferior lateral protocerebrum (ΔILPC–MB bilateral neuron). (**A,B**) Frontal and dorsal views of the stained neurons. In total, 5 neurons were labelled in the sample, of which 4 neurons showed bilateral innervation. All 5 neurons innervated the lateral ΔILPC. One neuron projected to the mushroom body calyx in the ipsilateral hemisphere. (**C**) Confocal stacks of neuronal innervation in the ipsilateral hemisphere. Most innervation was biased towards the lateral ΔILPC. The shape of neuropils is shown with a broken line. (**D**) Odour response profile of the neurons. The grey box represents the time period of odour presentation (500 ms).

Neurons innervating the MB calyx in the contralateral hemisphere (one unilateral neuron and four bilateral neurons, ∆ILPC–MB bilateral neuron) were labelled (Fig. 6). The innervation in the ∆ILPC was biased towards the lateral side in all labelled neurons (Fig. 6C). The cell bodies were located in the cell cluster between the midbrain and optic lobe, and the axon ran through the LALC and projected to the contralateral hemisphere. A recording of one of five stained neurons showed excitation in response to bombykal but not to bombykol (Fig. 6D).

In addition, we identified a neuron innervating the ∆ILPC, the SMP, and the medial lobe of the MB (Supplementary Fig. 1). The cell body was located on the anterior surface and medial to the AL macroglomerular complex (MGC). A smooth innervation was observed in the β and β′ lobelets but not in the γ and medial lobelets (Supplementary Fig. 1C). The area of neuronal innervation was consistent with the result of mass staining (Fig. 2E–G). The innervation in the SMP and ∆ILPC exhibited a blebby appearance, indicating a feedback pathway from the MB to the ∆ILPC and SMP. The neuron with a similar morphology to MB–SMP/∆ILPC is present in *Drosophila* (Gad1-F-800172, FlyCircuit Database)^17^.

### Areas connected with the LH identified by mass staining

**Figure 7:**
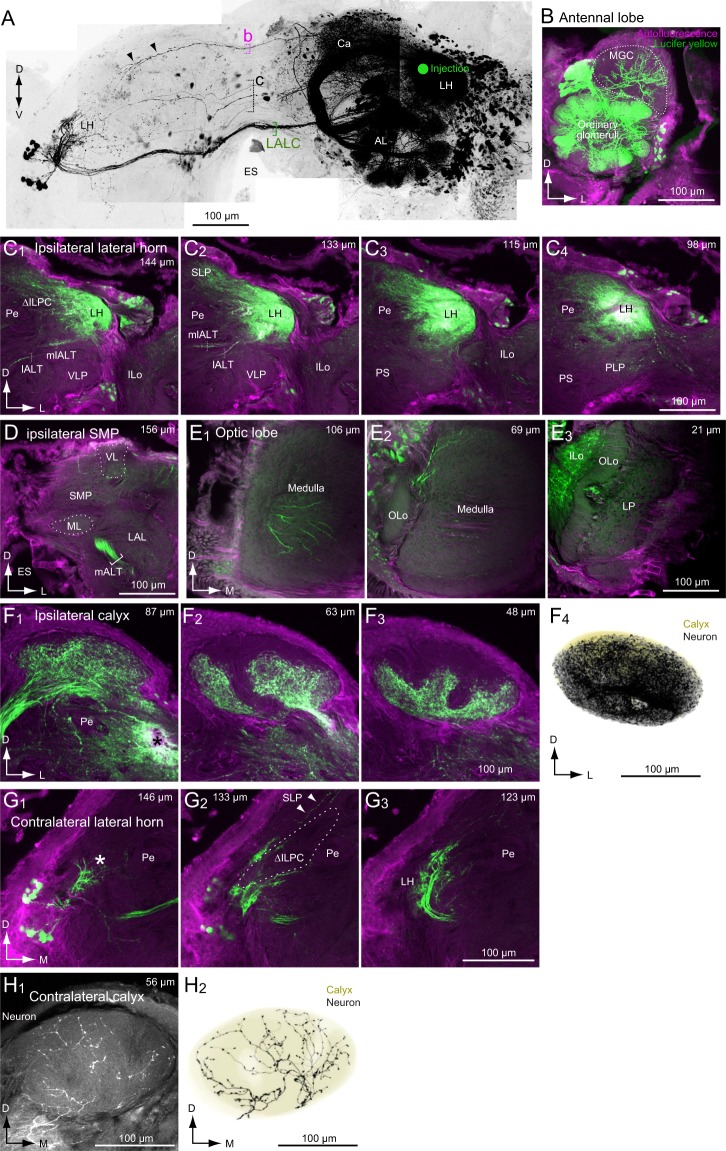
Mass staining into the delta area of the lateral horn (LH). (**A**) Maximum-intensity projection of the mass staining results. The green circle represents the site of dye injection into the LH. The original data are taken from^15^. (**B**) Confocal stack of the antennal lobe in the sample shown in (**A**). Ordinary glomeruli were densely labelled. The shape of the macroglomerular complex (MGC) is shown with a broken line. Green shows Lucifer yellow signals in stained neurons, and magenta shows the autofluorescence of the tissue. (**C**) Confocal stacks of the LH in the hemisphere ipsilateral to the injection site. Depth from the posterior surface is shown in the top-right panel. The superior lateral protocerebrum (SLP) was labelled. (**D**) Confocal stack of the SMP in the hemisphere ipsilateral to the injection site. No innervation was observed in the SMP. €: Confocal stacks of the optic lobe in the hemisphere ipsilateral to the injection site. Innervation was observed in the inner lobula (ILo). A few projections were observed in the medulla. (**F**) The mushroom body calyx in the hemisphere ipsilateral to the injection site. The anterior ventral area of the calyx was densely labelled. The reconstruction of neuronal innervation and the shape of the calyx are shown (E4). (**G**) Confocal stacks of the LH in the hemisphere contralateral to the injection site. The shape of the delta area of the inferior lateral protocerebrum (ΔILPC) is shown with a broken line. The LH and a small region anterior to ΔILPC were labelled (asterisk), whereas the ΔILPC was not labelled. A few projections were observed in the superior protocerebrum (SPC). (**H**) The mushroom body calyx in the hemisphere contralateral to the injection site. Reconstruction of neuronal innervation and the shape of the calyx are shown (H2). The innervation was sparse compared with that in the ipsilateral calyx. ES, esophagus; mALT, medial antennal-lobe tract; mlALT, medio-lateral antennal-lobe tract; ML, medial lobe of the mushroom body; LAL, lateral accessory lobe; lALT, lateral antennal-lobe tract; LP, lobula plate; OLo, outer lobula; Pe, pedunculus of the mushroom body; PLP, posterior lateral protocerebrum; PS, posterior slope; SMP, superior medial protocerebrum; VL, vertical lobe of the mushroom body; VLP, ventral lateral protocerebrum.

To investigate the connectivity of the LH, we injected dye into this region (Fig. 7). The superior lateral protocerebrum (SLP) (Fig. 7C) and anterior MB calyx were densely labelled, whereas few innervations were observed in the SMP (Fig. 7D), which was the primary target of the ∆ILPC (Figs 1 and 2). A few projections were observed in the medulla (Fig. 7E1,2), and the inner lobula in the ipsilateral optic lobe was labelled (Fig. 7E3). Ordinary glomeruli in the AL on the ipsilateral side were densely labelled, which was consistent with a known anatomical feature of PNs^3^. In addition, a sparse innervation was observed in the MGC (Fig. 7B), which may be the innervation of multiglomerular PNs, some of which send sparse projections to the ∆ILPC as well as the LH (Supplementary Fig. 2 for morphology).

Labelling was observed in the hemisphere that was contralateral to the injection site (Fig. 7A). The highest number of neurons crossed the midline through LALC (Fig. 7A). Another population of neurons ran through pathway “b”, which was observed following dye injection in the ∆ILPC (Fig. 1A). Some neurons crossed the midline through the deep brain region that was posterior to the central body (Fig. 7A, “c”). These axons ran through different routes and did not form a bundle. A bilateral projection was observed through the LALC to the LH (Fig. 7G), and some projections reached the MB calyx (Fig. 7H), whereas bilateral projections were observed through pathway “b” to the SLP (Fig. 7A, arrowheads). The innervation to the lateral protocerebrum was bifurcated into two projections: a minor projection to the region anterior to the ∆ILPC (Fig. 7G1) and a major projection to the LH in the region posterolateral to the ∆ILPC (Fig. 7G3).

### LH neurons

**Figure 8:**
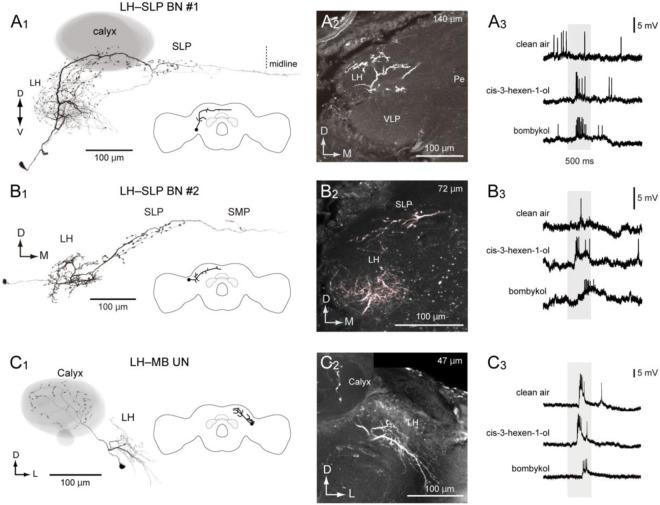
Morphology and physiology of a projection neuron from the lateral horn (LH). (**A**) LH–SLP bilateral neuron #1. Frontal view of the morphology of the neuron (**A1**). The innervation has a smooth appearance in the LH and a blebby appearance in the mushroom body calyx and superior lateral protocerebrum (SLP). The inset shows a schematic of the neuronal innervation. Neuronal innervation in the lateral protocerebrum is shown (**A2**). The depth from the posterior brain surface is shown in the top-right panel. (**A3**) Response profile of the neuron. The neuron exhibits an excitatory response with differential latency in response to odour and mechanosensory stimuli. **(B)** LH–SLP bilateral neuron #2. **(B1)** Frontal view of the morphology of the neuron. The innervation has a smooth appearance in the LH and a blebby appearance in the superior medial protocerebrum (SMP) and superior lateral protocerebrum (SLP). The inset shows a schematic of the neuronal innervation. (**B2**) Neuronal innervation in the LH. The innervation is biased towards the anterior LH. (**B3**) Response profile of the neuron. **(C)** LH–MB unilateral neuron. (**C1**) Frontal view of the morphology of the neuron. The mushroom body is shown in grey. The innervation has a smooth appearance in the LH and a blebby appearance in the calyx. The inset shows a schematic of the neuronal innervation. (**C2**) Confocal stacks of neuronal innervation in the lateral protocerebrum. (**C3**) Response profile of the neuron. The grey box represents the time period of odour presentation.

Because mass staining showed that the SLP was well connected to the LH, electrodes were also inserted into the SLP, located within a shallow depth in the brain. In total, we observed three stained neurons innervating the LH that were responsive to odour stimuli. We identified a couple of bilateral neurons that connected the LH and SLP in the ipsilateral hemisphere (Fig. 8A,B). One neuron innervated the entire field in the LH, and innervation with a smooth appearance was predominant (Fig. 8A). Blebby projections were observed in the SLP, and the axon crossed the midline through pathway “b.” Another neuron innervated the anterior LH (Fig. 8B). Both neurons exhibited an excitatory response with different response latencies to *cis*-3-hexen-1-ol and bombykol (Fig. 8A,B, right panel). We identified the LH–MB unilateral neuron connecting the LH and MB calyx (Fig. 8C). The innervation in the LH had a smooth appearance and that in the calyx had a blebby appearance. The projection covered wide areas in the calyx. The neuron exhibited transient excitation in response to olfactory stimuli.

## Discussion

We identified candidate pathways from the LPC to higher centres in the moth brain. Further, we characterized individual neuronal components constituting these pathways. Intracellular recording confirmed that these were olfactory neurons. We focused on the terminal morphology of individual neurons because it is a good predictor for post-synaptic dendrites and pre-synaptic axonal terminals^18–21^. A blebby or varicose appearance is likely to indicate post-synaptic terminals, whereas a smooth or fine appearance is likely to indicate pre-synaptic terminals^20^. We discuss the LPC output at the cellular level.

### Third-order centres: ∆ILPC to SMP/LH to SLP

We identified three bombykol-responsive neurons connecting the ΔILPC to the SMP, two bilateral and one unilateral. A neuron with a similar morphology to ∆ILPC–SMP BN has been reported in *Drosophila*^22,23^ (fru-F-300124, FlyCircuit Database^17^). Female *Drosophila* show attractive behaviour towards a sex pheromone. Kohl *et al*. (2013) identified *fruitless*-positive LH neurons in females termed aSP-g, which responded specifically to the *Drosophila* sex pheromone^22^. The dendrite of aSP-g overlaps the axon of DA1 PNs, which are sensitive to sex pheromone^24^. Similar to ∆ILPC–SMP BN in the silkmoth, these neurons send their axons to the SMP in both hemispheres. Another *fruitless*-positive LH neuron termed aSP-h shares basic anatomical features with ∆ILPC–SMP UN, although aSP-h selectivity is broad and is not specific to the sex pheromones. These findings indicate the general importance of the SMP in pheromone processing in insects. A recent connectomics study in *Drosophila* identified the SMP as a major target of the LH output neurons^25^. The superior intermediate protocerebrum and SMP are also well connected to the LH. Additionally, a neuron that responded specifically to a blend of pheromones and innervated the ∆ILPC and SMP has been reported in *Manduca sexta*^26^.

**Figure 9:**
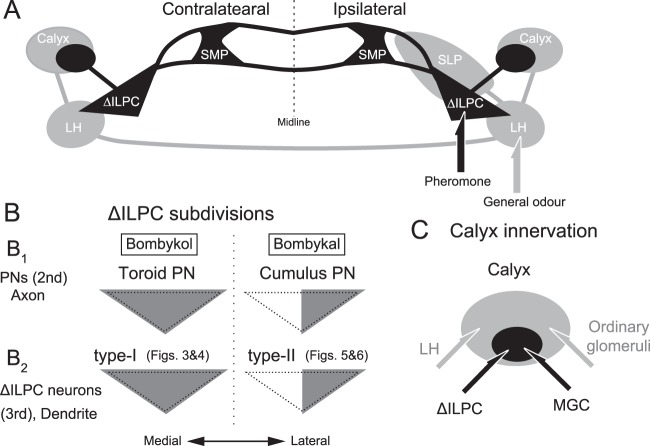
Summary diagram of the olfactory pathway in the protocerebrum. (**A**) Neuropils connected with the ∆ILPC and LH identified in the present study. Regions involved in pheromone and plant odour processing are shown with black and grey, respectively. (**B**) Neurite innervation in the ∆ILPC. Grey indicates the area of innervation. Axonal projection of PNs arising from the toroid and cumulus (top) and the dendritic arborization of ∆ILPC neurons are shown (bottom). (**C**) Spatial distribution of neuronal innervation to the calyx. Output neurons from the MGC and ∆ILPC project to the central zone (black), whereas those from ordinary glomeruli and the LH project to a wider area (grey).

In the silkmoth, the circuit for plant odour processing following the LH remains unclear^16^. We identified the LH output neuron projecting to the SLP (Fig. 8A,B) but not to the SMP, which is connected to the ∆ILPC. This suggests that the SLP processes plant odour information. These anatomical observations support the presence of parallel pathways for processing pheromone and plant odour information at the third-order neurons from the periphery (Fig. 9A). A similar parallel pathway for processing olfactory information is present in *Drosophila*^27^. There are two distinct populations of LH output neurons, each of which projects to the SMP and SLP.

### Subdivision in the ∆ILPC

We observed two types of spatial distribution in the smooth innervation of third-order neurons. In contrast to the terminal morphology of PNs with a blebby appearance^2^, the neurite of third-order neurons showed a smooth appearance in the ∆ILPC (Figs 3–6), suggesting that they were post-synaptic dendrites^18,19^. Some neurons innervate the entire ∆ILPC (type-I; Figs 3 and 4), whereas others innervate the lateral ∆ILPC (type-II; Figs 5 and 6).

A similar morphological feature is observed in axon projection by PNs. There are functional subdivisions for the target zone of PNs for different pheromone components. Toroid PNs project to the entire ∆ILPC, whereas cumulus PNs project to the lateral ∆ILPC^14^ (Fig. 9B1). A similar profile of PN axonal projection has been reported in the moth *Helicoverpa assulta*^28^, suggesting a common strategy for processing multiple pheromone components in moths. This anatomical observation captured the information flow of pheromone components. Neurons innervating the entire ∆ILPC exhibited excitation in response to bombykol (Figs 3 and 4, type-I), and those innervating the lateral ∆ILPC exhibited brief excitation to bombykal but not to bombykol (Figs 5 and 6, type-II). Thus, there exists a correspondence between the position of the dendritic field and pheromone responsiveness. This suggests the presence of different functional subdivisions for pheromone components in ∆ILPC output neurons, with each subdivision within the ∆ILPC possessing a preference for pheromone responsiveness (Fig. 9B2).

The presence of a segregated representation of pheromone components at the output level from the LPC is largely unknown in other insects. Using *in vivo* calcium imaging, Roussel *et al*. (2014) analysed the spatial pattern of neuronal activity in the honeybee LH^29^. The region responding to a pheromone overlaps the region responding to plant odours. Further, there are substantial overlaps among the activated regions for different pheromones for queen recognition, alarm, and aggregation. Therefore, in contrast to *Bombyx*, the pheromone representation in the third-order centre may not be segregated in honeybees.

The spatial distribution of PNs from different pheromonal glomeruli has been reported in the American cockroach^30^, which has two pheromonal glomeruli that are responsive to sex pheromones. PNs from both glomeruli project to the anterior LH, which has a triangular shape with a location reminiscent of ∆ILPC in *Bombyx*. The fields of axonal projection from these two glomeruli exhibit a high degree of overlap. The two glomeruli process different sex pheromones; however, both components elicit courtship behaviour. It is of interest to study whether segregation in dendritic innervation of third-order neurons is present in the cockroach.

### Pathway from the ∆ILPC/LH to the MB calyx

We characterized connections between LPC and the MB calyx using the mass staining technique and identified individual neuron morphology (Figs 5, 6 and 8). According to the terminal morphology, these neurons supply the signal from the ∆ILPC/LH to the calyx. The connection with the ∆ILPC was localized to the anterior central zone of the calyx (Fig. 1D,E), whereas that with the LH occupied a large volume in the calyx (Fig. 7F). This spatial relationship was similar to that of the axonal projection by PNs: PNs from pheromonal glomeruli project to the central zone, whereas PNs from ordinary glomeruli project to a wide field in the calyx^31^, suggesting that the spatial pattern of axonal projection of LH output neurons and PNs is shared (Fig. 9C).

Neurons connecting the LH and the MB calyx have been reported in other insects. Papadopoulou *et al*. (2011) identified a large GABAergic neuron in locusts, which has a smooth process in the peduncle, the vertical lobe of the MB, and the LH and has a blebby process in the calyx^32,33^. Takahashi *et al*. (2017) identified four types of giant interneurons in the cockroach, each of which has a smooth process in the LH and a blebby process in the calyx^34^. Furthermore, a putative homologous neuron has been identified in *Drosophila* (APL neuron)^35^. Axonal projections of the neurons mentioned above are distributed widely throughout the calyx with dense innervation. In contrast to the ∆ILPC–MB UN and LH–MB UN in *Bombyx* (Figs 5 and 8C), these neurons show a dense innervation and a dendritic field in the MB lobe. Such a convergent projection has been reported in the cockroach, where four types of neurons have innervations to different areas in the LH, but all project to the entire field within the calyx^34^. Gupta & Stopfer (2012) identified a population of an LH interneuron termed C1^32^, which showed morphological similarity to LH–MB UN in *Bombyx* (Fig. 8C). C1 has a smooth process in the LH and a blebby process in the calyx, and the innervation in the calyx is relatively sparse. Neurons that share morphological features with type-I UN are present in *Drosophila* (FlyCircuit Database: VGlut-F-400240, VGlut-F-800056, VGlut-F-600030, and VGlut-F-500419)^17^ and are candidates for homologous neurons. The innervation in the calyx was not dense and localized in the dorsal calyx.

## Conclusion

In the present study, we investigated third-order olfactory neurons by population and single-cell levels in the moth. We identified neuronal pathways from the LPC and characterized individual neurons. Future studies should examine the physiology of these neurons.

## Supplementary information

Supplementary Information
