# Supplementary material for: Morphology and physiology of olfactory neurons in the lateral protocerebrum of the silkmoth Bombyx mori
Source: Sci Rep. 2019 Nov 12;9:16604. doi: 10.1038/s41598-019-53318-8 (PMC6851382; doi:10.1038/s41598-019-53318-8)
Supplement: Supplementary file 1 — Supplementary Information [file 41598_2019_53318_MOESM1_ESM.docx]

**Scientific Reports**

**Supplementary Information**

**Morphology and physiology of olfactory neurons in the lateral protocerebrum of the silkmoth *Bombyx mori***

Shigehiro Namiki, Ryohei Kanzaki

Research Center for Advanced Science and Technology, The University of Tokyo, 4-6-1 Komaba, Meguro, Tokyo 153-8904, Japan. Correspondence should be addressed to S.N. (email: [namiki@rcast.u-tokyo.ac.jp](mailto:namiki@rcast.u-tokyo.ac.jpr))

2 Supplementary Figures


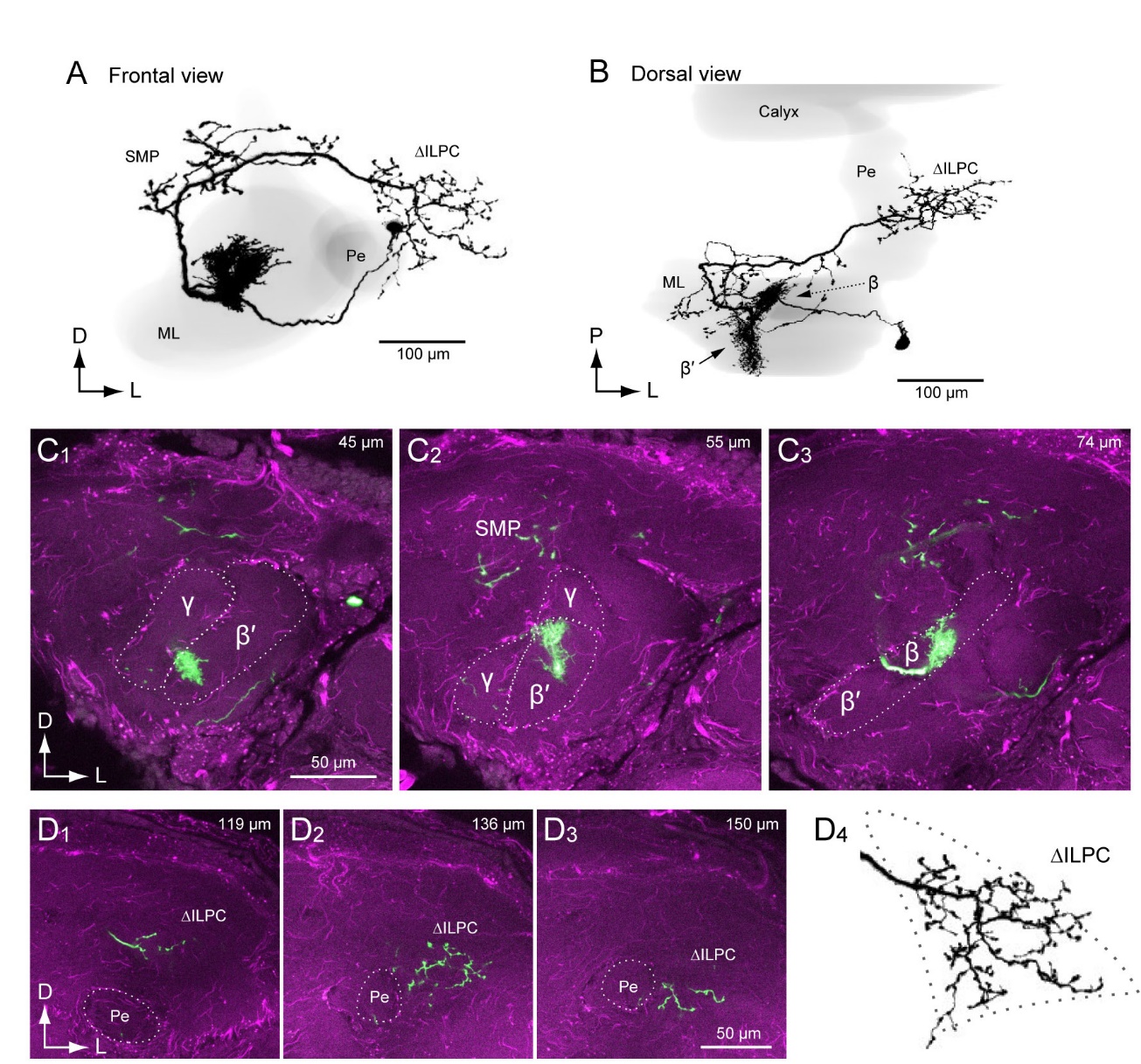


**Supplementary Fig. 1. Morphology of a neuron innervating the medial lobe, superior medial protocerebrum, and the delta area of the inferior lateral protocerebrum (MB–ΔILPC/SMP neuron)** (**A,B**): Frontal and dorsal views of the reconstruction of the neuron. The innervation has a smooth appearance in the medial lobe and blebby appearance in SMP and ΔILPC. The cell body is located dorsomedial to the antennal lobe. The shape of the mushroom body is shown in yellow. (**C**): Confocal stacks of the neuronal innervation in the medial lobe of the mushroom body. Tufted branches are present in β and β′ lobelets. The neuron does not innervate the γ lobelet. The depth from the anterior surface of the protocerebrum is shown in in the top-right panel. The shape of the lobelets is shown with a broken line. (**D**): Confocal stacks of the neuronal innervation in ΔILPC (D1-D3). Three-dimensional reconstruction of the neuronal innervation is shown (D4). The neuron innervates the entire ΔILPC. The shape of ΔILPC is shown with a broken line.


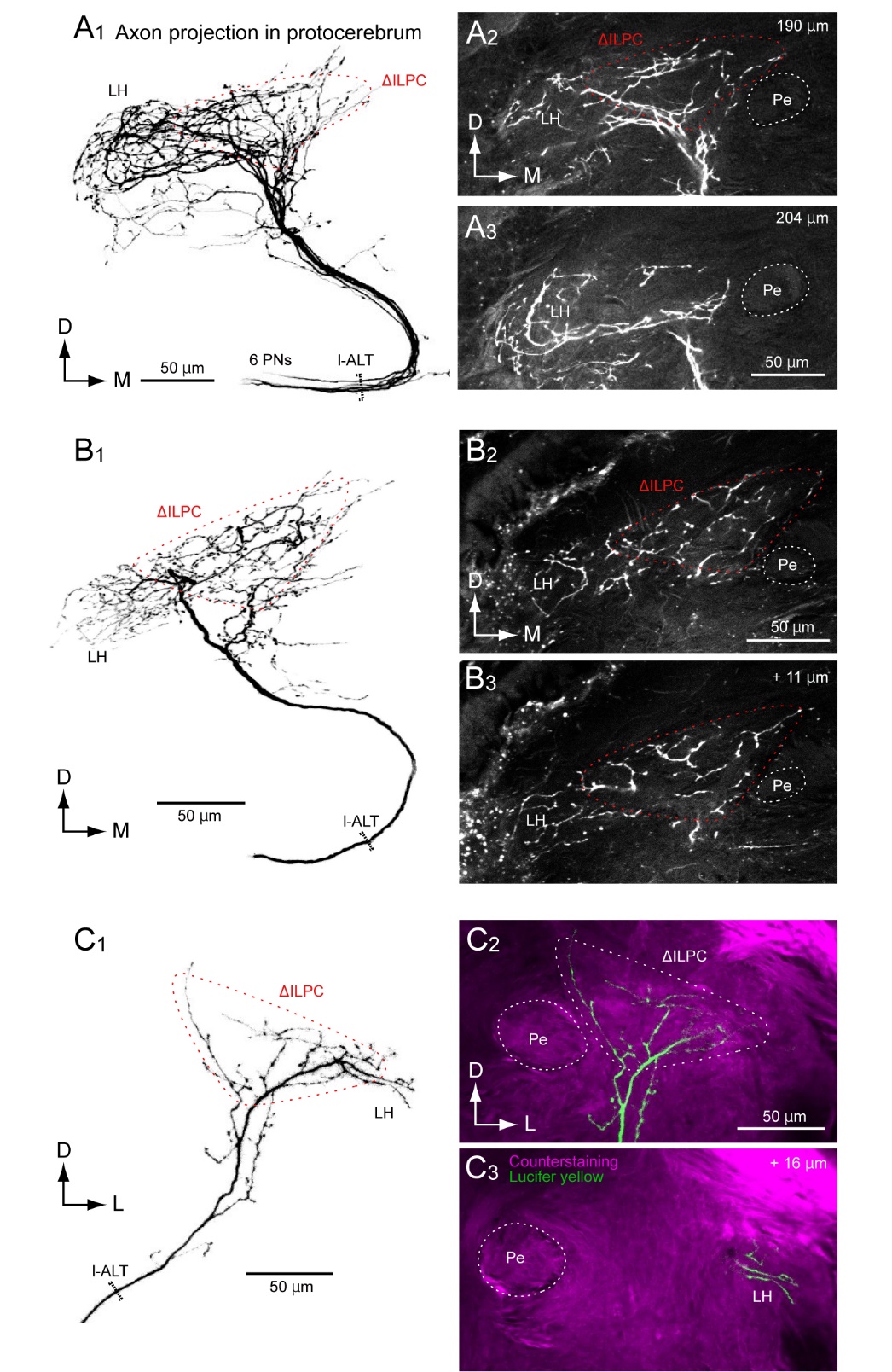


**Supplementary Fig. 2. Axonal projection of the multiglomerular projection neurons.** (**A-C**): Three examples of axonal projection of projection neurons running through the lateral antennal-lobe tract (l-ALT). Maximum intensity projection (*left*) and confocal stacks are shown (right). The results of mass staining are shown in panel A. In total, 6 projection neurons were labeled, each of which arborize both cumulus and toroid in the macroglomerular complex. The depth from the anterior brain surface is shown in the top-right panel. The results of single-neuron staining are shown in panels B and C. The distance between the stacks is shown in the top-right panel. In all cases, the neurons have innervation to the lateral horn. LH, lateral horn; Pe, pedunculus of the mushroom body.
